# Research Trends on Pesticide Exposure and Cancer Development: A Global Literature Review (2005–2024)

**DOI:** 10.3390/ijerph23040493

**Published:** 2026-04-14

**Authors:** Murugappan Sivagami, Robin Haunschild

**Affiliations:** 1 Rathinam College of Arts and Science (Autonomous), Rathinam Techzone Campus, Eachanari, Coimbatore 641021, Tamil Nadu, India; sivagami.library@rathinam.in; 2 Max Planck Institute for Solid State Research, Heisenbergstr 1, 70569 Stuttgart, Germany

**Keywords:** pesticide exposure, bibliometric analysis, cancer, neoplasm

## Abstract

**Highlights:**

**Public health relevance—How does this work relate to a public health issue?**

- This study systematically analyzes global research linking pesticide exposure with cancer, a major public health concern affecting agricultural workers and exposed populations.
- By identifying dominant and emerging research themes, the study clarifies how scientific attention has evolved toward health risks associated with pesticide use.

**Public health significance—Why is this work of significance to public health?**

- The findings demonstrate a clear shift in research focus from occupational exposure assessment to toxicity and cancer-related health outcomes, reflecting growing concern over long-term population health effects.
- Mapping research hotspots and gaps provides evidence to support more targeted public health surveillance and risk assessment strategies.

**Public health implications—What are the key implications or messages for practitioners, policy makers and/or researchers in public health?**

- The results support the need for improved regulatory oversight and occupational health protections for populations exposed to pesticides.
- Identified research trends can guide policy makers and researchers in prioritizing preventive interventions and future studies addressing pesticide-related cancer risks.

**Abstract:**

This study examines the global research landscape on the relationship between pesticide exposure and cancer using bibliometric and scientometric approaches. A total of 3908 records published between 2005 and 2024 were retrieved from the Web of Science database using an elaborate search strategy incorporating pesticide-related keywords (e.g., atrazine, glyphosate, DDT, chlorpyrifos) and general cancer descriptors (cancer and neoplasm). The analysis explores publication trends, citation patterns, keyword co-occurrence, and co-citation networks to understand the evolution of research in this field. The results reveal a consistent increase in publication output, indicating growing global attention to pesticide-related health risks. Keyword burst analysis and temporal thematic assessment highlight a clear evolution in research focus, shifting from early studies on occupational exposure and epidemiological risk assessment toward recent emphasis on toxicity, oxidative stress, and mechanistic pathways underlying carcinogenesis. The findings provide important insights for future research, public health policy, and regulatory frameworks, emphasizing the need for interdisciplinary approaches. By identifying emerging themes and research gaps, this study offers a broad understanding of the development of pesticide–cancer research and supports efforts to mitigate the health impacts of pesticide exposure.

## 1. Introduction

The potential association between pesticide exposure and cancer development has received increasing attention in epidemiological and toxicological research. Several studies have reported associations between pesticide exposure and various cancer outcomes, including non-Hodgkin lymphoma, breast cancer, lung cancer, and brain tumors [1,2,3,4,5]. These findings have raised concerns regarding possible health risks among populations with occupational or environmental exposure to pesticides, particularly agricultural workers and residents of farming regions [6].

Previous studies have examined the health impacts and carcinogenic potential of pesticides from different perspectives. For instance, Giambò et al. [7] reported that several major pesticide categories exhibit carcinogenic potential. Similarly, Alavanja et al. and Bassil [1,8] identified associations between elevated pesticide exposure and increased cancer incidence. Environmental contamination studies have also highlighted potential risks; for example, Panis et al. [9] reported that pesticide contamination of drinking water may increase cancer risk in certain regions. Other studies have explored broader health implications of pesticide exposure. Valcke et al. [10] emphasized that the health benefits of fruit and vegetable consumption generally outweigh the risks associated with pesticide residues [11], while Freeman [12] demonstrated that pesticides may influence gene expression and induce epigenetic alterations.

In addition to epidemiological and toxicological investigations, scientometric and bibliometric approaches have been increasingly used to examine the development of scientific fields [13]. For example, Mao et al. [14] analyzed research trends related to pesticides and neurological diseases using keyword co-occurrence analysis, while Chen et al. [15] applied bibliometric techniques to examine the development of research literature in environmental studies. Similarly, Sweileh [3] used social network analysis to map research on health impairments associated with agricultural pesticide exposure. These studies demonstrate the usefulness of scientometric methods for understanding research development and identifying knowledge structures within scientific literature.

As the volume of research on pesticide exposure and cancer continues to grow, it becomes important to systematically analyze the evolution of this research field [16]. Scientometric tools such as CiteSpace, Pajek, and VOSviewer enable the visualization of knowledge networks and the identification of research trends, thematic developments, and collaboration patterns within scientific literature [17,18,19,20]. Techniques such as keyword co-occurrence and citation network analysis help reveal major research themes, emerging topics, and the intellectual structure of a scientific domain [21,22].

However, despite the growing number of studies examining pesticide exposure and cancer, a comprehensive scientometric overview of the global research landscape in this field remains limited. Therefore, the present study aims to examine the global research landscape on the relationship between pesticide exposure and cancer development using scientometric techniques. By analyzing publications indexed in the Web of Science Core Collection from 2005 to 2024, the study explores publication trends, keyword patterns, and collaboration networks to provide a systematic overview of the development of research on pesticide exposure and cancer. Unlike conventional bibliometric studies that primarily rely on descriptive mapping, the present study integrates temporal thematic evolution with structural network analysis to provide a dynamic understanding of pesticide–cancer research.

## 2. Research Methodology

### 2.1. Scientometric Approach

This study applies scientometric methods to quantitatively examine the development of research on pesticide exposure and cancer. Scientometric analysis allows the identification of research trends, collaboration patterns, and emerging topics within a scientific field through the analysis of bibliographic data [23].

In this study, Bibexcel 2020 was used for statistical data processing and bibliographic data management. CiteSpace 6.3.1 was used for network visualization and analysis to identify knowledge structures, research hotspots, and evolving scientific trends through co-citation and keyword co-occurrence analysis [21]. The analysis was conducted using time slicing from 2005 to 2024 with one-year intervals. Node types included keywords and cited references, and the top 50 most frequent items from each time slice were selected. The Pathfinder pruning algorithm was applied to simplify the network structure.

The dataset includes epidemiological, toxicological, and experimental studies addressing pesticide exposure and cancer outcomes. However, the present analysis focuses on bibliometric characteristics of the literature rather than comparing individual study designs.

### 2.2. Data Source and Search Strategy

The Web of Science Core Collection (Clarivate Analytics) was selected as the primary data source because it provides high-quality indexed journals, standardized bibliographic information, and reliable citation data widely used in bibliometric and scientometric studies.

The search focused on studies related to pesticide exposure and cancer published between 2005 and 2024. The search query was developed through an iterative process to capture both general pesticide exposure terms and specific pesticide compounds frequently discussed in epidemiological and environmental health research.

The search query used was:

TS = ((pesticide exposure OR pesticides exposure OR atrazine OR glyphosate OR dichlorodiphenyldichloroethylene OR dichlorodiphenyltrichloroethane OR chlorpyrifos OR carbofuran OR organochlorine OR pendamethalin OR pendimethalin) AND (cancer OR neoplasm OR neoplasms)) AND PY = 2005–2024.

The search strategy also incorporated commonly studied pesticide classes such as organophosphates, organochlorines, and carbamates frequently reported in the literature. The selected pesticide compounds represent widely investigated chemicals in environmental and occupational exposure studies, ensuring that the search captured major research topics within the field.

The search strategy combined pesticide-related terms with cancer-related terms to capture studies examining the potential relationship between pesticide exposure and cancer outcomes. In addition to specific pesticide compounds, broader pesticide class terms were included to capture studies addressing different categories of pesticide exposure.

After applying the search query, 3908 records were retrieved from the database and used for subsequent bibliometric and network analyses. The dataset was directly exported from the Web of Science and analyzed using bibliometric tools such as CiteSpace, which include basic built-in preprocessing functions. No manual data cleaning or keyword standardization was performed.

### 2.3. Eligibility Criteria

To ensure the relevance and quality of the dataset, specific inclusion and exclusion criteria were applied. Studies were included if they addressed pesticide exposure, cancer development, or the association between pesticide exposure and cancer (according to the search query to ensure reproducibility), were published between 2005 and 2024, were indexed in the Web of Science (WoS) Core Collection. WoS has a bias toward indexing papers published in English. However, we did not restriction of the dataset to publications written in English.

### 2.4. Data Processing and Analysis

The retrieved records were exported from the Web of Science database and imported into Bibexcel for data processing and preliminary statistical analysis. Microsoft Excel 2019 was used for data organization, cleaning, descriptive statistical analysis, and graphical representation.

CiteSpace software was used to construct and visualize knowledge networks, including collaboration networks, keyword co-occurrence networks, and co-citation networks. In these visualizations, nodes represent entities such as authors, institutions, countries, or keywords, while links between nodes represent relationships such as collaboration, co-occurrence, or co-citation [24]. The size of nodes reflects the number of publications or frequency of occurrence, while the color of nodes indicates the publication year.

### 2.5. Social Network Analysis

Social network analysis has been widely used to examine scientific collaboration patterns and knowledge structures in research fields [12,24]. To examine the structural characteristics of the research networks, several network centrality measures were used. Degree centrality measures the number of direct connections a node has with other nodes in the network. In this study, a higher degree centrality indicates that an author or institution collaborates with a larger number of partners within the research field. Betweenness centrality identifies nodes that act as intermediaries connecting different clusters within the network. Nodes with high betweenness centrality play an important role in facilitating knowledge exchange between different research groups. Closeness centrality reflects how easily a node can interact with all other nodes in the network based on the shortest paths. A higher closeness centrality suggests that a node is well positioned to disseminate information efficiently across the network. Sigma, a metric used in CiteSpace, is calculated as (betweenness centrality + 1) × citation burst. High sigma values indicate nodes that are structurally significant and associated with emerging or innovative research directions. Together, these network measures help identify influential actors, emerging research themes, and structural relationships within the field of pesticide exposure and cancer research [25].

Disparate conventional bibliometric studies that primarily rely on descriptive indicators such as publication counts and co-occurrence networks, the present study integrates keyword co-occurrence, citation burst detection, and temporal thematic evolution analysis to provide a multi-dimensional understanding of the research landscape. This approach enables the identification of not only dominant research themes but also their dynamic progression over time, thereby offering a broader analytical framework.

## 3. Results

### 3.1. Temporal Evolution of Publications and Citations

**Figure 1:**
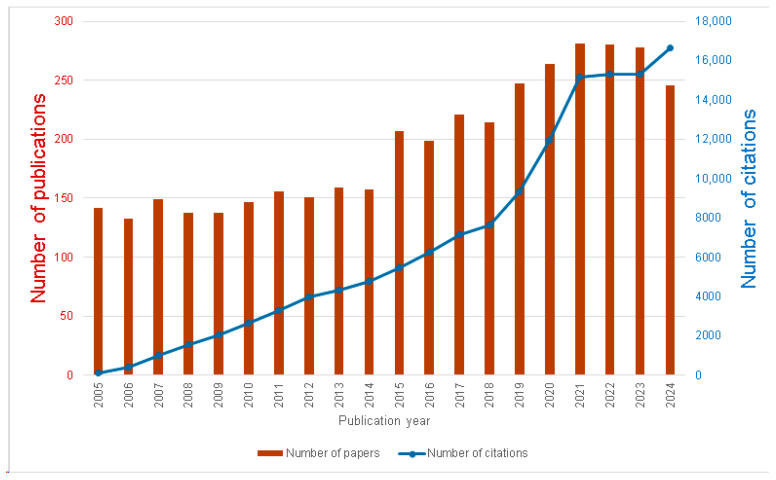
Temporal Evolution of Annual Publication Output and Citations.

The temporal evolution of publications and citations in the field from 2005 to 2024 is shown in Figure 1. Both the annual publication output and the number of citations per year show an overall upward trajectory. The publication output starts with 142 publications in 2005 which gradually increases to its highest point of 281 in 2021, with a similar level (280 and 278) sustained in 2022 and 2023. A slight decline to 246 publications in 2024 might be due to incomplete indexing at the time of data retrieval 20 August 2025. The number of citations linearly increases between 2005 and 2018. The period between 2018 and 2021 is characterized by a strong increase in the number of citations whereas we observe a plateau-like development between 2021 and 2023. Finally, the number of citations significantly increases from 2023 to 2024. This overall growth of publication output and number of citations reflects a continuous increase in research interest in the domain.

Table 1 presents the frequency distribution of the most prominent keywords across different time intervals, offering a clear overview of the thematic evolution within pesticide–cancer research. The results indicate a gradual shift in research focus, reflecting both continuity and the emergence of new scientific priorities.

Certain keywords exhibit distinct temporal patterns. Some topics appear consistently across all periods, suggesting their foundational importance, whereas others demonstrate phase-specific growth. For instance, “oxidative stress” shows a marked increase in the most recent period (2021–2024), indicating a growing emphasis on understanding the biological and mechanistic pathways underlying pesticide-induced carcinogenesis.

Similarly, the increasing frequency of “occupational exposure” and “agriculture” highlights a strengthened research focus on human–environment interactions, particularly concerning occupational health risks among agricultural workers. This trend reflects rising global awareness of exposure-related health implications.

Moreover, the emergence and growth of keywords such as “glyphosate” and “risk assessment” demonstrate a shift toward chemical-specific investigations and regulatory concerns, aligning with contemporary debates on pesticide safety and public health. Overall, the table reveals a clear diversification of research themes over time, transitioning from general associations between pesticides and cancer to more specialized, mechanism-driven, and policy-relevant studies, thereby illustrating the field’s responsiveness to evolving scientific and societal challenges.

### 3.2. Country, Institution, and Journal Analysis

**Figure 2:**
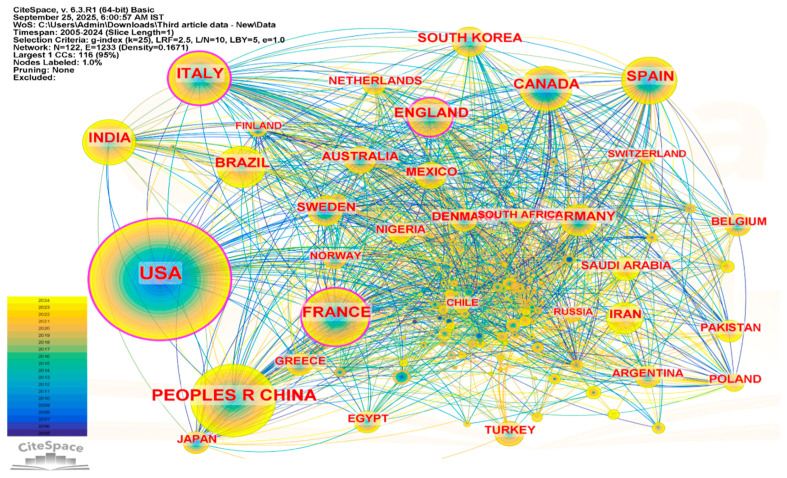
Country-level collaboration network with countries from the institutional affiliation of authors contributing to the publications.

Figure 2 illustrates the country-level collaboration network in pesticide exposure and cancer research. The visualization highlights patterns of international collaboration among countries contributing to this research field. The colored rings around nodes represent the temporal distribution of publications (blue–green–yellow indicating earlier to more recent publications), while pink rings indicate nodes with higher betweenness centrality, which may suggest a bridging position in the collaboration network [25].

The United States appears as the largest node in the network, reflecting the highest number of publications (1212) within the dataset. It also shows relatively high betweenness centrality (0.26), suggesting that it occupies an important connecting position within the international collaboration structure. China is the second most productive country with 481 publications; however, its lower centrality value (0.10) indicates a more limited bridging position within the global co-authorship network despite its high publication output. Several other countries, including Italy (0.14), England (0.12), and France (0.11), also exhibit notable centrality values, indicating that they may serve as intermediary nodes connecting different groups of collaborating countries. Countries such as Canada, Spain, Brazil, and Sweden show moderate levels of collaboration within the network, contributing to the overall structure of international research cooperation.

Citation burst analysis reflects periods in which publications from specific countries received increased attention in the literature. In this dataset, relatively strong citation bursts are observed for the United States, Iran, and Brazil, followed by Canada, Saudi Arabia, Nigeria, Sweden, Japan, Spain, and Turkey. These bursts may indicate periods of increased research activity or growing attention to studies from these countries. The collaboration network consists of 167 nodes and 759 links, with a network density of 0.0273, suggesting the presence of a moderately connected international collaboration structure. Overall, the network indicates active global participation in pesticide exposure and cancer research, although further strengthening of collaboration among countries, particularly across developing regions, may contribute to a more integrated research landscape.

**Figure 3:**
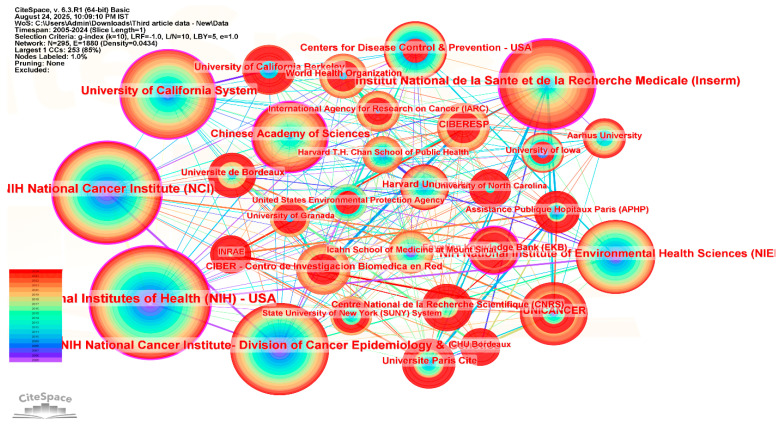
Cooperation Network on the Basis of Institutions.

Figure 3 presents the institutional collaboration network in pesticide exposure and cancer research. The network consists of several interconnected institutions that contribute actively to this research area. Larger nodes represent institutions with a higher number of publications, while links between nodes indicate collaborative relationships among institutions.

Several major biomedical and public health research institutions appear prominently in the network. Institutions such as the National Institutes of Health (NIH, USA), the National Cancer Institute (NCI, USA), the Division of Cancer Epidemiology and Genetics, INSERM (France), and the Centers for Disease Control and Prevention (USA) are represented by larger nodes and multiple collaborative links, reflecting their substantial publication activity and involvement in institutional collaborations within the dataset.

Betweenness centrality, which reflects the position of an institution within the collaboration network, is relatively high for the NCI Division of Cancer Epidemiology and Genetics (0.21). Other institutions with notable centrality values include INSERM (0.17), Aarhus University (0.16), University of California Berkeley (0.14), and NIH–NIEHS (0.13). These values suggest that these institutions occupy intermediary positions that may facilitate connections among different institutional clusters in the collaboration network.

The sigma values reported by CiteSpace represent a combination of betweenness centrality and citation burst indicators. Institutions such as the NCI Division of Cancer Epidemiology and Genetics, INSERM, Aarhus University, University of California Berkeley, and NIEHS show sigma values of 1.00, indicating nodes that combine structural network positioning with periods of increased research attention within the dataset.

Overall, the collaboration network shows active participation from research institutions in North America and Europe, with additional contributions from institutions in other regions such as the Chinese Academy of Sciences and the Egyptian Knowledge Bank. The presence of institutions from multiple regions suggests an expanding international research collaboration landscape in studies related to pesticide exposure and cancer.

**Figure 4:**
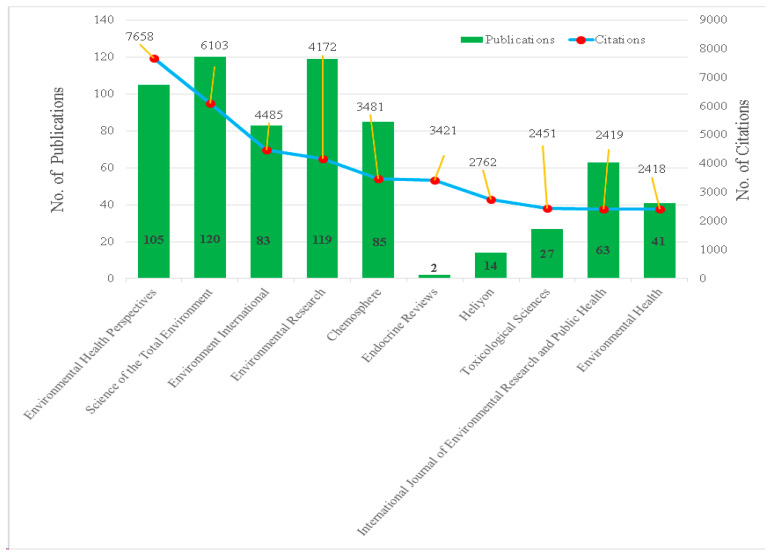
Major Journals in Terms of Citation Frequency. The Line Connecting the Points is Intended to Only Guide the Eye.

Figure 4 demonstrates the journal-wise distribution of publications and citations reveals a well-defined stratification between high-volume dissemination outlets and high-impact knowledge carriers within the field. Science of the Total Environment (120 publications; 6103 citations) and Environmental Research (119 publications; 4172 citations) emerge as the most prolific journals, indicating their role as major platforms for sustained and wide-ranging dissemination of environmental research. In contrast, Environmental Health Perspectives combines substantial productivity (105 publications) with the highest citation impact (7658 citations), underscoring its position as the field’s most influential and authoritative journal. Environment International (83 publications; 4485 citations) and Chemosphere (85 publications; 3481 citations) also demonstrate a strong balance between output and impact, reflecting their importance in advancing interdisciplinary environmental science and exposure-related research. A striking pattern of citation intensity is observed for Endocrine Reviews, which, despite contributing only two publications, accrues 3421 citations, highlighting the exceptional influence of review-based, mechanism-oriented scholarship. Heliyon (14 publications; 2762 citations) exhibits moderate productivity with relatively high visibility, while Toxicological Sciences (27 publications; 2451 citations) maintains a focused yet impactful presence within toxicology. International Journal of Environmental Research and Public Health (63 publications; 2419 citations) and Environmental Health (41 publications; 2418 citations) further reinforce the growing integration of environmental research with public health and policy-relevant perspectives. Overall, the distribution reflects a mature and diversified publication ecosystem in which high-output multidisciplinary journals ensure broad dissemination, while specialized and review-oriented journals disproportionately shape the intellectual and citation impact of the field.

### 3.3. Analysis of Most Cited References

The most cited references have received most attention and can be considered to be very influential publications in a particular research field. Such publications usually are very relevant for upcoming researchers to contribute their work. However, it is to be kept in mind that older publications have an advantage over younger publications when comparing raw citation counts. Table 2 lists the ten most cited references with their local citation counts (i.e., within the studied dataset). The most cited reference, Guyton et al. (2015, 61 citations) [19], is pivotal as it presents the classification by the International Agency for Research on Cancer for glyphosate, diazinon, and malathion as “probably carcinogenic to humans,” sparking global debate and influencing regulatory policies worldwide. Andreotti et al. (2018, 52 citations) [26], based on the large US Agricultural Health Study, provides robust cohort evidence on glyphosate use and cancer risk, reporting no significant overall association but noting suggestive links with acute myeloid leukemia, thereby offering nuanced insight into exposure–disease relationships. Benbrook et al. (2016, 44 citations) [27] tracks four decades of glyphosate usage, revealing its dramatic global increase after the adoption of genetically modified crops, highlighting environmental and health concerns related to long-term and large-scale exposure. Alavanja et al. (2003, 43 citations) [28] was among the earliest to demonstrate an association between pesticide exposure and prostate cancer risk, setting a precedent for later epidemiological studies. Sung et al. (2021, 39 citations) [29] is broader in scope and provides GLOBOCAN 2020 global cancer statistics which serve as an essential reference for contextualizing pesticide-related cancer risks within worldwide cancer burdens. Koutros et al. (2013, 38 citations) [30] narrows the focus to aggressive prostate cancer and reinforces concerns about pesticide-related occupational exposures. Kim et al. (2017, 37 citations) [31] synthesizes epidemiological and toxicological evidence and offers a broad review of pesticides’ effects on human health, which is widely used as a reference framework in subsequent studies. Weichenthal et al. (2010, 36 citations) [16] systematically reviews cancer incidence from the Agricultural Health Study cohort, strengthening the evidence base by pooling long-term findings. Sharma et al. (2019, 35 citations) [32] broadens the perspective by analyzing worldwide pesticide usage trends and their ecological and health impacts, bridging environmental science and public health. Finally, Bonner et al. (2016, 34 citations) [33] provides valuable insights into occupational exposure and lung cancer incidence, a relatively less-studied outcome compared to prostate cancer, thus diversifying the research scope. Together, these studies not only emphasize the central role of glyphosate and other commonly used pesticides but also underscore occupational risks, epidemiological evidence from large cohorts, and broader global health and environmental concerns, forming the backbone of scientific and policy discussions in this area.

### 3.4. Co-Citation Analysis

**Figure 5:**
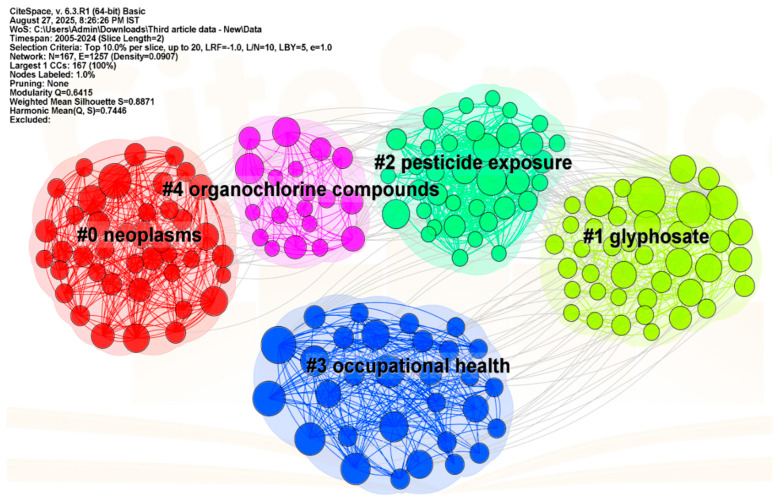
Topic Cluster Network of a Co-Citation Analysis.

The co-citation clustering analysis presented in Table 3 and Figure 5 identifies the five major research themes (topics) linking pesticide exposure to cancer. Cluster #0 (Cancer Incidence: Neoplasm, avg. year 2002) is the oldest and centers on large cohort studies such as the US Agricultural Health Study which established epidemiological evidence for the link between pesticide exposure and increased risks for prostate, breast, and lung cancers. Cluster #1 (Glyphosate-based Herbicide, avg. year 2014) reflects the intense global debate around glyphosate, triggered by IARC’s classification of glyphosate as “probably carcinogenic” and subsequent toxicological and regulatory studies exploring its mechanisms, its potential for DNA damage, and its links to multiple types of cancer. Cluster #2 (Prostate Cancer Risk: Pesticide Exposure, avg. year 2009) reinforces prostate cancer as a critical endpoint, with studies showing dose–response relationships and strengthening occupational exposure evidence. Cluster #3 (Prostate Cancer: Occupational Health, avg. year 2019) represents more recent research, integrating mechanistic studies with cohort findings to emphasize workplace pesticide hazards in prostate cancer risk, reflecting ongoing concern in occupational epidemiology. Finally, Cluster #4 (Organochlorine Compound, avg. year 2006) focuses on persistent pesticides such as DDT, documenting long-term health effects including endocrine disruption and cancer risk, often traced through breast milk and environmental pathways. Together, these clusters illustrate the evolution from broad cancer incidence studies to chemical-specific debates (glyphosate, organochlorines) and occupational cancer risks, showing how evidence has expanded from general associations to mechanistic and regulatory implications.

**Figure 6:**
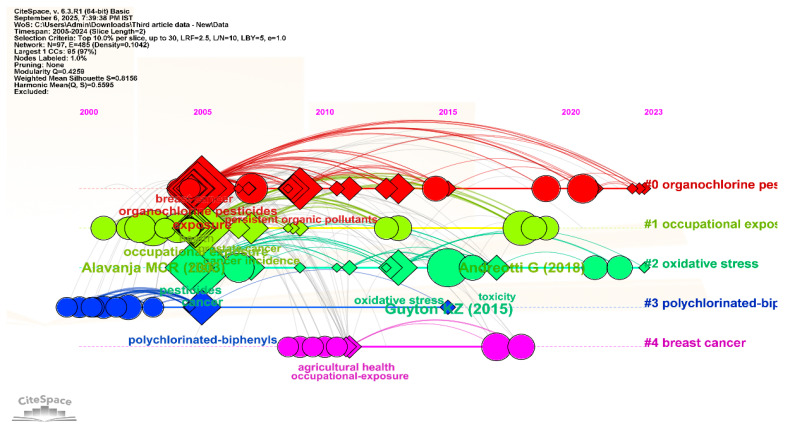
Timeline of Co-Citation Document Clusters from 2005 to 2024 the three references in the figure are [19,26,28].

Figure 6 presents the timeline visualization of reference keywords, illustrating the thematic evolution and structure of research on pesticide exposure and cancer. The network consists of 97 nodes and 485 links, where nodes represent keywords and links indicate co-occurrence relationships within the literature. Diamond-shaped nodes denote keywords with relatively high degree centrality (≥15), suggesting that these terms are connected with multiple research topics, while circular nodes represent keywords with higher citation frequencies (≥20), indicating commonly referenced concepts within the dataset. The clusters are identified using log-likelihood ratio (LLR) analysis, which groups related keywords based on shared citation patterns and thematic similarity.

Cluster 0 is the largest cluster, containing 30 keywords with a silhouette score of 0.733, indicating reasonable cluster consistency. This cluster primarily focuses on research related to organochlorine pesticide contamination and environmental exposure. The literature in this cluster frequently addresses topics such as pesticide residues in environmental media and their potential associations with cancer-related outcomes.

Cluster 1 includes 27 keywords with a silhouette score of 0.825, suggesting a well-defined thematic grouping. This cluster represents studies examining occupational exposure to pesticides, particularly among agricultural workers and other populations with frequent contact with pesticide chemicals. Research within this theme often investigates exposure patterns, occupational risk factors, and related epidemiological observations.

Cluster 2 contains 17 keywords and has a silhouette score of 0.833, reflecting strong thematic coherence. The cluster highlights studies exploring molecular and biochemical mechanisms associated with pesticide exposure, with particular attention to oxidative stress and cellular responses reported in toxicological and biomedical research.

This cluster includes 11 keywords and has a high silhouette value of 0.96, indicating a clearly defined thematic group. The research in this cluster focuses on polychlorinated biphenyls (PCBs) and related persistent organic pollutants, examining their environmental persistence and potential associations with cancer-related health outcomes.

Cluster 4 consists of 9 keywords with a silhouette score of 0.851. The cluster highlights research exploring breast cancer in relation to environmental or pesticide-related exposures, including studies examining epidemiological patterns and possible interactions between environmental factors and disease outcomes.

Overall, the clusters represent several interconnected thematic areas within the literature, including environmental contamination, occupational exposure, molecular mechanisms, persistent pollutants, and specific cancer types. The timeline structure illustrates how these research themes have developed and interacted over time within the broader field of pesticide exposure and cancer studies. Together, the clusters provide a structured overview of the main topics explored in the scientific literature and demonstrate the multidisciplinary nature of research in this area.

Keyword analysis helps identify major research themes, emerging topics, and evolving research priorities within a scientific field. By examining the co-occurrence and frequency of keywords, it is possible to understand how research on pesticide exposure and cancer has developed over time and to detect shifts in scientific focus.

**Figure 7:**
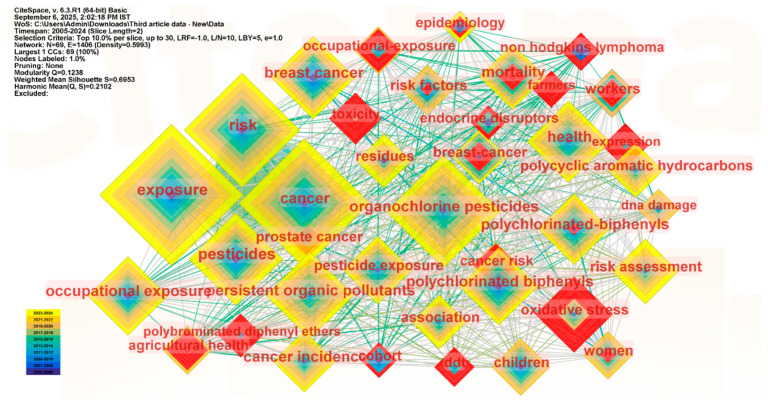
Network of Co-Occurring Keywords with Citation Burst (Red Filled Nodes).

Figure 7 illustrates the keyword co-occurrence network representing the thematic structure of research on pesticide exposure and cancer. In this network, nodes represent keywords extracted from the literature, while links indicate the frequency with which two keywords appear together in the same publications. The size of a node reflects the frequency of keyword occurrence, and the connections between nodes illustrate relationships among research topics.

Several frequently occurring keywords are observed in the network, including Exposure, Risk, Cancer, Pesticides, Organochlorine Pesticides, Polychlorinated Biphenyls, Occupational Exposure, Mortality, Health, and Breast Cancer. The frequent appearance of these terms suggests that studies in the dataset commonly address topics related to environmental and occupational exposure to pesticides and associated health outcomes.

Keywords such as Occupational Exposure, Farmers, and Workers appear within the network as terms associated with studies examining exposure in occupational settings. Similarly, chemical-specific terms including Organochlorine Pesticides, Polychlorinated Biphenyls, Persistent Organic Pollutants, and DDT indicate the presence of research focusing on particular groups of pesticide compounds frequently discussed in the literature.

Disease-related keywords such as Breast Cancer, Prostate Cancer, and Cancer Risk represent research topics that explore associations between pesticide exposure and specific cancer types. Their occurrence in the network suggests that these disease categories have received attention within the broader research field.

Centrality measures within the network indicate that keywords such as Exposure and Risk occupy relatively central positions in the co-occurrence structure, reflecting their frequent connections with other keywords in the dataset. These terms therefore appear as commonly shared concepts linking multiple research themes.

Overall, the keyword co-occurrence network highlights several interconnected thematic areas within the literature, including exposure assessment, pesticide compounds, occupational exposure contexts, and cancer-related health outcomes. The network structure provides an overview of the main topics addressed in the research field and illustrates how different themes are connected within the scientific literature.

### 3.5. Research Trend Analysis

Keyword burst analysis was conducted to trace research hotspots and their temporal evolution [18]. Table 4 presents the top 20 keywords with the strongest citation bursts, clearly illustrating shifts in research priorities over time. The most prominent burst is observed for Toxicity (strength = 22.59; 2017–2024), indicating a sustained recent emphasis on understanding the adverse biological effects of pesticide exposure. Agricultural Health also shows a strong burst (19.85; 2011–2018), reflecting increasing concern for health risks among farming communities. Early high-intensity bursts such as Organochlorine Compounds, Farmers, Workers, and Risk-Factors highlight the field’s initial focus on occupational exposure and persistent pesticides. Keywords related to cancer outcomes, including Breast Cancer Risk, Breast-Cancer, and Non-Hodgkins Lymphoma, demonstrate continued epidemiological interest in pesticide-associated carcinogenesis. Genotoxicity shows a short-duration burst, suggesting a temporary research emphasis rather than a continuing or dominant theme. In more recent years, bursts in Oxidative Stress and Expression indicate a growing shift toward mechanistic investigations addressing cellular and molecular responses to pesticide exposure. Overall, the burst pattern reveals a clear transition from exposure- and occupation-centered studies toward research emphasizing toxicity mechanisms, cancer risk, and broader human health implications.

The temporal thematic evolution analysis in Table 5 reveals a clear and systematic progression in pesticide–cancer research, reflecting the maturation of the field from descriptive exposure assessment to mechanistic understanding. In the initial phase (2005–2010), research was predominantly centered on identifying sources of pesticide exposure and environmental contamination, with particular emphasis on agricultural settings and persistent organic pollutants such as organochlorines and DDT. This phase corresponds to a foundational stage in which the primary objective was to establish potential links between pesticide exposure and adverse health outcomes, largely driven by occupational and environmental health concerns.

The second phase (2010–2018) marks a significant transition toward epidemiological and population-based investigations. During this period, research expanded to examine associations between pesticide exposure and specific cancer types, including breast cancer, leukemia, and other malignancies. The increasing prominence of terms such as “risk assessment,” “epidemiology,” and “cancer risk” indicates a shift from exposure identification to quantifying disease burden and establishing statistical correlations. This phase reflects the consolidation of evidence linking pesticide exposure to carcinogenic outcomes, supported by large-scale cohort and case–control studies.

In the most recent phase (2018–2024), the research focus has further evolved toward mechanistic and molecular-level investigations. The emergence of keywords such as “oxidative stress,” “toxicity,” “epigenetics,” and “endocrine disruption” highlights a paradigm shift toward understanding the biological pathways underlying pesticide-induced carcinogenesis. This transition suggests that the field has moved beyond establishing associations to exploring causal mechanisms at the cellular and genetic levels. Such developments are indicative of a more advanced stage of scientific inquiry, where interdisciplinary approaches integrating toxicology, molecular biology, and environmental science are increasingly employed.

Overall, the observed progression from exposure assessment to epidemiological validation and finally to mechanistic exploration underscores a logical and cumulative development of knowledge in pesticide–cancer research. This evolution not only reflects advancements in analytical techniques and data availability but also aligns with growing global concerns regarding pesticide safety and regulatory policies. Importantly, our findings highlight a continuing need to integrate mechanistic insights with epidemiological evidence to strengthen causal inference and support evidence-based public health interventions.

**Figure 8:**
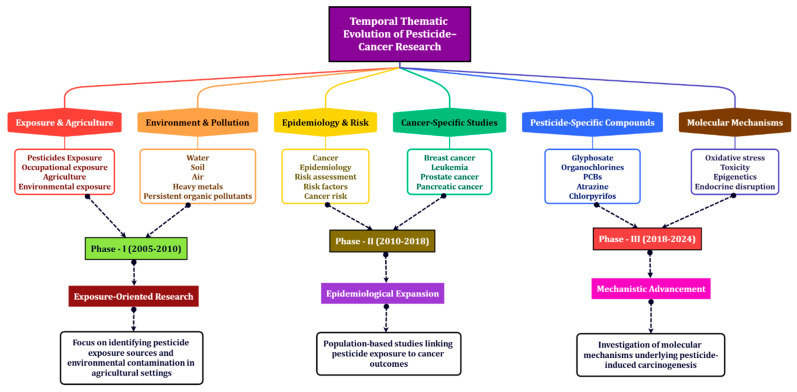
Conceptual Framework of the Evolution of Pesticide-Cancer Research.

The conceptual framework presented in Figure 8 illustrates the unified temporal framework provides a broader understanding of how pesticide–cancer research has evolved over time. The evolution is structured into three major phases, each representing a distinct stage in the development and maturation of the research field.

The first phase, exposure-oriented research (2005–2010), is characterized by a strong emphasis on identifying sources of pesticide exposure and environmental contamination. Research during this period primarily focused on agricultural settings, occupational exposure, and persistent pollutants such as organochlorines. The prominence of environmental and exposure-related themes indicates that early investigations were largely exploratory, aiming to establish the presence and extent of potential health risks associated with pesticide use.

The second phase, epidemiological expansion (2010–2018), reflects a shift toward population-based and disease-oriented research. During this period, studies increasingly examined associations between pesticide exposure and specific cancer types, including breast cancer, leukemia, and prostate cancer [31,34]. The integration of epidemiological methods and risk assessment approaches signifies a transition from descriptive exposure studies to analytical investigations aimed at quantifying cancer risk and establishing statistical relationships. This phase represents a critical stage in consolidating empirical evidence linking pesticide exposure to carcinogenic outcomes.

The third phase, mechanistic advancement (2018–2024), demonstrates a further progression toward understanding the underlying biological processes of pesticide-induced carcinogenesis. Research in this phase is dominated by themes such as oxidative stress, toxicity, epigenetics, and endocrine disruption, indicating a strong focus on molecular and cellular mechanisms [35]. This shift highlights the increasing adoption of interdisciplinary approaches, combining toxicology, molecular biology, and environmental science to explore causal pathways and biological plausibility.

Overall, Figure 8 highlights a coherent and cumulative progression of knowledge, moving from exposure identification to epidemiological validation and finally to mechanistic explanation. This evolution reflects not only advancements in research methodologies and analytical tools but also a growing emphasis on translating scientific findings into evidence-based public health and regulatory strategies. The observed thematic progression is consistent with the co-citation structure of the field, further validating the transition from foundational exposure studies to advanced mechanistic research. This unified temporal framework provides a broader understanding of how pesticide–cancer research has evolved over time.

## 4. Discussion

The findings of the present study provide an overview of the intellectual and thematic structure of pesticide–cancer research. The co-citation and keyword co-occurrence analyses reveal the major research domains and their interconnections, consistent with previous bibliometric studies that have mapped the structure of scientific knowledge [18,21]. However, beyond these conventional outputs, the present study integrates multiple analytical approaches to provide a more nuanced understanding of the field.

In particular, the incorporation of keyword burst analysis enables the identification of emerging research fronts, reflecting the dynamic nature of scientific development [18,21]. The results indicate a clear shift in research focus over time, with recent emphasis on topics such as oxidative stress, toxicity, and molecular-level mechanisms.

Rather than reiterating individual findings, the integrated analysis highlights a consistent and progressive shift across multiple dimensions of the research landscape. Unlike conventional bibliometric studies that primarily rely on descriptive indicators, this approach provides a structured interpretation of how research themes evolved over time [22]. This enhances the analytical depth of the study by moving beyond static mapping toward a dynamic understanding of knowledge development.

The results demonstrate a clear transition from exposure-oriented research to epidemiological investigations and, more recently, to mechanistic and molecular-level studies. This progression reflects the increasing sophistication of research methodologies and the growing emphasis on understanding causal pathways linking pesticide exposure to cancer outcomes [21]. Such a shift indicates the maturation of the field from descriptive assessments to more advanced explanatory research.

Furthermore, the integration of multiple bibliometric techniques in this study provides complementary insights into the research landscape. While co-citation analysis reveals the intellectual base and foundational studies within the field, keyword-based temporal analysis captures the evolving thematic focus [14]. This multi-dimensional approach strengthens the overall analytical framework and addresses limitations associated with single-method bibliometric studies.

The findings also have important implications for future research and policy. The increasing focus on mechanistic pathways, such as oxidative stress and endocrine disruption, highlights the need for interdisciplinary research integrating toxicology, molecular biology, and environmental science. Such integration is essential for strengthening evidence-based risk assessment and informing regulatory decisions related to pesticide exposure and public health [23].

At the same time, the study underscores the importance of cautious interpretation of bibliometric indicators. Citation-based measures and network centrality reflect structural patterns within the literature rather than direct measures of scientific impact or causality. Therefore, the results should be interpreted as indicative of research trends and knowledge structures rather than definitive evidence of causal relationships [26].

Overall, the study contributes to the existing literature by providing an integrated analytical framework that combines structural, temporal, and thematic dimensions of pesticide–cancer research. This approach enhances the understanding of the field’s development and provides a basis for identifying future research directions and knowledge gaps. These findings should be interpreted in light of the study’s methodological and data-related limitations.

## 5. Limitations

This study has several limitations that should be acknowledged. First, the search strategy was deliberately designed using general cancer descriptors (“cancer” and “neoplasm”) to capture a broad overview of pesticide–cancer research. While this approach reduces bias toward specific cancer subtypes, it may have excluded studies that primarily use more specialized terminology (e.g., leukemia, lymphoma, or melanoma). Similarly, the inclusion of selected pesticide-related terms represents a balance between search simplicity and dataset coverage, which may have led to the omission of some relevant publications.

Second, the analysis is based solely on the Web of Science (WoS) database. Although WoS ensures high-quality and standardized indexing, it does not provide complete global coverage, particularly for publications from the Global South and non-English sources. As a result, certain regional research contributions may be underrepresented.

Third, the study relies on bibliometric indicators such as citation counts, burst strength, and centrality measures, which reflect structural and relational patterns within the scientific literature rather than direct measures of research quality, scientific influence, or causal relationships. These indicators should therefore be interpreted with caution.

Furthermore, keyword-based analyses, including frequency and co-occurrence methods, depend on author-provided terms and may not fully capture the conceptual complexity or thematic diversity of the research field. Although built-in preprocessing functions were applied, variations in terminology may still influence the results.

Finally, while the temporal thematic evolution framework provides a structured interpretation of the progression of research themes, it is based on aggregated keyword patterns and does not explicitly model citation-based knowledge flows, as in main path analysis. Future research could integrate multiple databases and advanced network-based approaches to further enhance the robustness and depth of analysis. These findings should be interpreted in light of the study’s methodological and data-related limitations.

## 6. Conclusions

The analysis highlights that research on pesticide exposure and cancer has grown steadily over the past two decades, reflecting an increasing global awareness of the health risks associated with pesticide use. The consistent rise in publication output indicates that scientists are actively working to understand the pathways through which pesticides contribute to cancer, particularly among vulnerable populations such as agricultural workers. At the same time, the fluctuations in citation trends suggest that while earlier studies have had time to establish their impact, newer research is still emerging and gaining recognition within the academic community. The exploration of keywords with strong citation bursts demonstrates that the focus of research has evolved from documenting exposure risks and epidemiological associations to investigating molecular mechanisms like oxidative stress. This shift underscores a broader and more nuanced understanding of how pesticides affect biological systems and contribute to long-term health consequences. The clustering of co-cited references and the network of interconnected keywords reveal a field that is increasingly interdisciplinary, integrating toxicology, environmental science, occupational health, and epidemiology to address the complexity of pesticide-related cancer risks. Overall, the findings emphasize the importance of sustained research efforts, collaborative approaches, and knowledge-sharing platforms to enhance scientific understanding and inform policy decisions. Continued investigation into both exposure pathways and underlying biological mechanisms will be essential for developing effective interventions and safeguarding public health. The trajectory of this research underscores its relevance and urgency, as communities and researchers alike strive to mitigate the harmful effects of pesticide use and improve health outcomes worldwide. A key contribution of this study lies in the integration of temporal thematic evolution with conventional bibliometric techniques, enabling a dynamic interpretation of research development beyond static descriptive analysis.

## 7. Implications

The findings from this study have significant implications for research, public health policy, and regulatory frameworks concerning pesticide exposure and cancer risk. The analysis of 3908 records over the last two decades’ highlights both the growing scientific attention and the persistent knowledge gaps in understanding how pesticides contribute to cancer development. The increasing volume of publications reflects heightened awareness among researchers, while keyword bursts and citation trends reveal shifting priorities from documenting exposure pathways to investigating molecular mechanisms and long-term health impacts. For researchers, the results underscore the importance of interdisciplinary collaboration, particularly between environmental scientists, toxicologists, epidemiologists, and public health experts. The emerging focus on oxidative stress, expression, and toxicity of exposure points to new avenues for exploring disease pathways and preventive strategies. The identification of vulnerable populations, such as agricultural workers, further calls for targeted investigations that incorporate occupational and demographic factors.

For policymakers and regulatory agencies, the study provides evidence that sustained monitoring and stricter controls on pesticide use are essential for mitigating cancer risks. The findings also highlight the need for updating safety guidelines, conducting long-term cohort studies, and promoting safer alternatives to high-risk pesticides. Public health initiatives can benefit from these insights by developing educational campaigns, workplace interventions, and health screenings tailored to populations most at risk. Overall, the study contributes to a deeper understanding of the scientific landscape surrounding pesticide exposure and cancer. By identifying key research trends and gaps, it offers a roadmap for future investigations and evidence-based decision-making, aimed at reducing the harmful impact of pesticides on human health and improving environmental safety.

## Figures and Tables

**Table 1:** Temporal Evolution of the Most Used Keywords with Absolute Values of the Left and Percentages on the Right.

Keywords/Period	2005–2008	2009–2012	2013–2016	2017–2020	2021–2024	Growth Curve	2005–2008	2009–2012	2013–2016	2017–2020	2021–2024	Growth Curve
Pesticides	107	103	110	145	189	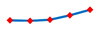	4.72	4.11	3.55	3.33	3.38	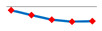
Cancer	36	47	39	53	63	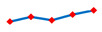	1.59	1.87	1.26	1.22	1.13	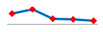
Breast Cancer	28	25	27	46	44	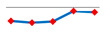	1.24	1.00	0.87	1.06	0.79	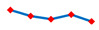
Pesticide	18	24	29	39	57	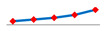	0.79	0.96	0.94	0.90	1.02	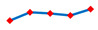
Organochlorine Pesticides	9	27	40	36	50	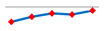	0.40	1.08	1.29	0.83	0.89	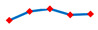
Risk Assessment	13	17	35	41	55	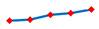	0.57	0.68	1.13	0.94	0.98	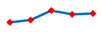
Epidemiology	26	41	23	27	36	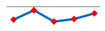	1.15	1.64	0.74	0.62	0.64	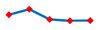
Glyphosate	4	2	12	56	65	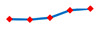	0.18	0.08	0.39	1.29	1.16	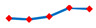
Occupational Exposure	26	18	16	28	32	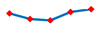	1.15	0.72	0.52	0.64	0.57	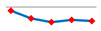
Polychlorinated Biphenyls	16	25	25	27	22	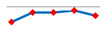	0.71	1.00	0.81	0.62	0.39	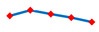
Agriculture	22	20	13	22	30	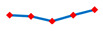	0.97	0.80	0.42	0.51	0.54	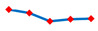
DDT	18	22	18	23	12	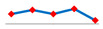	0.79	0.88	0.58	0.53	0.21	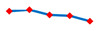
Persistent Organic Pollutants	9	9	25	24	21	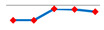	0.40	0.36	0.81	0.55	0.38	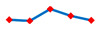
Oxidative Stress	2	4	14	16	44	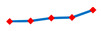	0.09	0.16	0.45	0.37	0.79	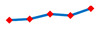
Exposure	9	11	13	23	21	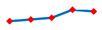	0.40	0.44	0.42	0.53	0.38	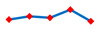
Total count of all keywords	2266	2507	3100	4350	5598							

**Table 2:** Top Ten Most Cited References.

Rank	Cited Reference	Author/Year	Citation Counts (Local)
1	Carcinogenicity of tetrachlorvinphos, parathion, malathion, diazinon, and glyphosate	Guyton, K.Z. et al. (2015), [19]	61
2	Glyphosate use and cancer incidence in the agricultural health study	Andreotti, G. et al. (2018), [26]	52
3	Trends in glyphosate herbicide use in the United States and globally	Benbrook, C.M. et al. (2016), [27]	48
4	Use of agricultural pesticides and prostate cancer risk in the Agricultural Health Study cohort	Alavanja, M.C.R. et al. (2003), [28]	45
5	Global cancer statistics 2020: GLOBOCAN estimates of incidence and mortality worldwide for 36 cancers in 185 countries	Sung, H. et al. (2021), [29]	44
6	Risk of total and aggressive prostate cancer and pesticide use in the Agricultural Health Study	Koutros, S. et al. (2013), [30]	42
7	Exposure to pesticides and the associated human health effects	Kim, K.H. et al. (2017), [31]	40
8	A review of pesticide exposure and cancer incidence in the Agricultural Health Study cohort	Weichenthal, S. et al. (2010), [16]	37
8	Worldwide pesticide usage and its impacts on ecosystem	Sharma, A. et al. (2019), [32]	37
10	Occupational exposure to pesticides and the incidence of lung cancer in the agricultural health study	Bonner, M.R. et al. (2016), [33]	36

**Table 3:** Topic Cluster Information from co-citation analysis.

# Cluster ID	Size	Silhouette	Average Year	Name (LLR ^1^)	Description
0	42	0.901	2002	Cancer Incidence: Neoplasm	The association between pesticide exposure and the incidence of multiple cancers, particularly prostate, breast, and lung neoplasms. The research trajectory highlights large-scale cohort studies such as the Agricultural Health Study, which provide epidemiological evidence linking occupational pesticide exposure to increased human cancer risk.
1	40	0.805	2014	Glyphosate-based Herbicide	The intense scientific and regulatory debate surrounding glyphosate, the world’s most widely used herbicide. Research highlights its potential carcinogenicity, with epidemiological studies linking exposure to cancers such as breast, prostate, and lung, while toxicological assessments explore mechanisms of DNA damage and endocrine disruption.
2	36	0.888	2009	Prostate Cancer Risk: Pesticide Exposure	Associations between occupational pesticide exposure and prostate cancer, with cohort data revealing dose–response patterns and strengthening evidence of environmental contributions to cancer risk.
3	30	0.950	2019	Prostate Cancer: Occupational Health	The role of occupational pesticide exposure in prostate cancer, supported by mechanistic and cohort studies that link workplace hazards to elevated cancer risk.
4	19	0.928	2006	Organochlorine Compound	Health effects of persistent organochlorine pesticides such as DDT, with studies linking exposure through breast milk and the environment to endocrine disruption and elevated cancer risk.

^1^ Log-likelihood ratio.

**Table 4:** Top 20 Keywords with the Strongest Citation Bursts.

Serial No	Keywords	Year	Strength	Begin	End	2005–2024
1	Organochlorine Compounds	2005	18.84	2005	2012	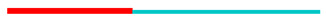
2	Farmers	2005	17.94	2005	2014	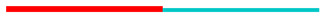
3	Risk-Factors	2005	15.3	2005	2010	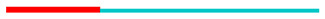
4	Breast Cancer Risk	2005	14.92	2005	2008	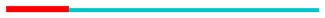
5	Workers	2005	14.88	2005	2012	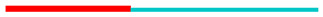
6	In Vitro	2005	12.7	2005	2008	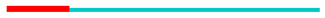
7	Mortality	2005	9.36	2005	2012	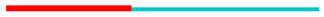
8	United-States	2005	8.68	2005	2006	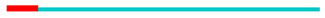
9	Breast-Cancer	2005	10.44	2007	2014	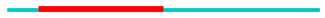
10	Population	2009	15.51	2009	2012	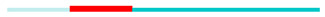
11	Agricultural Health	2011	19.85	2011	2018	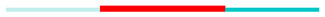
12	Non Hodgkins Lymphoma	2005	9.92	2011	2014	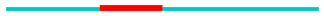
13	Polybrominated Diphenyl Ethers	2009	14.46	2013	2020	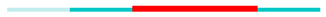
14	Toxicity	2017	22.59	2017	2024	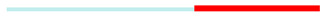
15	Genotoxicity	2017	10.49	2017	2018	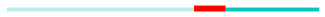
16	Expression	2013	9.8	2017	2024	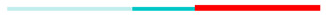
17	Oxidative Stress	2007	10.68	2019	2024	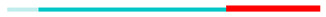
18	Human Health	2019	9.12	2019	2020	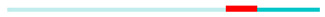
19	DDT	2011	8.9	2019	2022	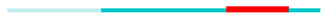
20	Cancer Risk	2007	8.77	2019	2022	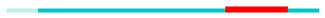

Note: In the timeline visualization, the blue shades represent periods without significant citation burst activity, indicating baseline or normal research attention. Darker blue tones reflect relatively lower intensity, while lighter blue tones indicate comparatively higher but still non-burst activity. The purple (or red-highlighted) segments represent periods of strong citation bursts, marking phases of intensified research focus and increased scholarly attention on the respective keyword.

**Table 5:** Temporal Thematic Evolution of Pesticide–Cancer Research.

Phase	Time Period	Dominant Research Focus	Key Themes Included	Representative Keywords	Scientific Interpretation
Phase 1	2005–2010	Exposure and environmental contamination	Exposure, Agriculture, Environmental pollution, Early chemical studies	Pesticides, DDT, Organochlorines, Occupational exposure, Environment	Early research focused on identifying sources of pesticide exposure and environmental contamination, particularly in agricultural settings
Phase 2	2010–2018	Epidemiological and disease association studies	Epidemiology, risk assessment, specific cancers	Cancer, Epidemiology, Breast cancer, Leukemia, Risk factors	Research expanded toward population-level studies examining associations between pesticide exposure and various cancer types
Phase 3	2018–2024	Mechanistic and molecular investigations	Toxicology, biological mechanisms, advanced chemical studies	Oxidative stress, Toxicity, Epigenetics, Endocrine disruption	Recent studies focus on biological and molecular mechanisms underlying pesticide-induced carcinogenesis

## Data Availability

We used proprietary data from WoS (Clarivate) which we are not allowed to share publicly. We will share the data upon reasonable request in accordance with the license agreement with Clarivate.
